# Delayed Ileal Perforation With Psoas Abscess and Fistulization Into the Inferior Vena Cava Post‐Dilation and Curettage—A Case Report

**DOI:** 10.1002/ccr3.71951

**Published:** 2026-02-08

**Authors:** Faiza Farooq, Bilal Aslam, Muhammad Hamza, Muhammad Saeed, Khalil El Abdi, Fareena Ambreen, Abdul Eizad Asif, Fazeela Bibi, Youssef Dadouche, Zaid Saimeh, Umama Alam, Said Hamid Sadat

**Affiliations:** ^1^ Radiology Department University of Lahore Teaching Hospital Lahore Pakistan; ^2^ Department of Medicine University of Lahore Lahore Pakistan; ^3^ Department of Medicine Saidu Medical College Swat Pakistan; ^4^ Department of Surgery D. G. Khan Medical College Dera Ghazi Khan Pakistan; ^5^ Faculty of Medicine and Pharmacy of Rabat Mohammed V University Rabat Morocco; ^6^ Khyber Girls Medical College Peshawar Pakistan; ^7^ Shalamar Medical and Dental College Lahore Pakistan; ^8^ Department of Medicine Jinnah Medical and Dental College Karachi Pakistan; ^9^ University of Jordan Amman Jordan; ^10^ Department of Medicine Khyber Medical College Peshawar Pakistan; ^11^ Kabul University of Medical Sciences Abu Ali Ibn Sina Kabul Pakistan

**Keywords:** dilation and curettage (D&C), ileal perforation, inferior vena cava (IVC) fistula, post‐D&C complications, psoas abscess

## Abstract

Dilation and curettage (D&C) is a common gynecological procedure, but it carries a risk of rare and catastrophic complications. We report the case of a 24‐year‐old female who presented with a 3‐month history of persistent low‐grade fever and progressive abdominopelvic pain following a D&C for a spontaneous miscarriage. Initial evaluations were inconclusive. A subsequent contrast‐enhanced computed tomography (CECT) scan revealed extensive bilateral psoas abscesses and, critically, the pathognomonic finding of free air within the lumen of the inferior vena cava (IVC), confirming an enterovascular fistula. An emergency exploratory laparotomy identified a contained ileal perforation with fistulous tracts extending to the right psoas muscle and the IVC. The patient was successfully managed with segmental ileal resection, abscess drainage, and primary suture repair of the IVC fistula. This case highlights a life‐threatening, delayed iatrogenic complication of uterine instrumentation and underscores that the radiological finding of intravascular gas is a surgical emergency, mandating immediate intervention to prevent fatal sepsis or systemic air embolism.

## Key Clinical Message

Persistent symptoms after dilation and curettage (D&C) warrant high suspicion for occult bowel injury. If perforation is suspected intraoperatively, 24‐h observation is prudent. The finding of intravascular gas on imaging confirms an enterovascular fistula—a surgical emergency requiring immediate intervention to prevent fatal sepsis or air embolism.

## 1 Introduction

Dilation and curettage (D&C) is a common and generally safe gynecological procedure. However, uterine perforation is a known complication, with a reported incidence of 0.3% to 2.6%, and can be associated with iatrogenic injury to adjacent structures, most often the ileum and sigmoid colon [1, 2]. While a significant bowel injury is typically recognized intraoperatively or presents acutely with peritonitis, a delayed presentation from a contained perforation is far more insidious and diagnostically challenging. Such an injury may evolve over weeks to months, leading to complex septic sequelae like intra‐abdominal or retroperitoneal abscesses [3].

An enterovascular fistula, representing a direct communication between the gastrointestinal tract and the major vessels, is an exceptionally rare and life‐threatening manifestation of this process, risking catastrophic sepsis or systemic air embolism. The development of a fistula directly into the inferior vena cava (IVC) following a gynecological procedure is a near‐fatal event [3].

Herein, we report a unique case of a 24‐year‐old female who presented 3 months after a D&C with the complete triad of a contained ileal perforation, extensive bilateral psoas abscesses, and a fistula into the inferior vena cava. This report aims to highlight this catastrophic, delayed complication of uterine instrumentation and underscore the critical role of cross‐sectional imaging in diagnosing an enterovascular fistula—a finding that mandates emergent surgical intervention.

## 2 Case History

A 24‐year‐old primigravida female with no significant past medical history presented to our hospital with a 3‐month history of persistent, non‐specific abdominal pain and fever. The patient's clinical course is summarized in Table 1. Four months prior to presentation, she experienced a spontaneous first‐trimester miscarriage that was managed with a suction D&C which was reported as uncomplicated in the available procedural records.

**TABLE 1:** Chronological summary of the patient's clinical history, detailing the key events, findings, interventions, and outcomes from the inciting dilation and curettage (D&C) through diagnosis, surgical management, and recovery.

Time point	Event	Key clinical findings and interventions
~4 months prior to admission	Dilation & curettage (D&C)	Performed for a spontaneous first‐trimester miscarriage; procedure noted as “uncomplicated” in available records
~1 week post‐D&C	Onset of symptoms	Patient developed intermittent low‐grade fever (up to 38.3°C) and progressive, dull aching pain in the lower abdomen and right flank
1 week to 3 months post‐D&C	Outpatient evaluation	Symptoms worsened; patient was evaluated for suspected ureteric colic, but an ultrasound was inconclusive.
Day 0	Hospital admission	Admitted with worsening pain, fever (38.1°C), tachycardia (110 bpm), and marked leukocytosis (18,500 cells/μL). A positive right psoas sign was elicited on examination
Day 1	Definitive diagnosis and emergency surgery	A CECT scan revealed bilateral psoas abscesses and the critical finding of air within the inferior vena cava (IVC). An emergency laparotomy was performed for segmental ileal resection, abscess drainage, and primary IVC repair
Post‐op Days 1–10	Inpatient recovery	The patient was monitored in the ICU for 48 h. Her fever and leukocytosis resolved within 3 days. She was discharged home on post‐operative day 10 to complete a course of oral antibiotics
6 months post‐operatively	Follow‐up assessment	The patient was asymptomatic with full functional recovery. A follow‐up Doppler ultrasound confirmed complete resolution of all abscess collections and normal patency of the IVC

Approximately one week after the D&C, she developed an intermittent low‐grade fever (up to 38.3°C), accompanied by a progressive, dull, aching pain in the lower abdomen and right flank. The pain radiated to her right lower back and anterior thigh and was exacerbated by walking. During this 3‐month period, she was evaluated at an outpatient clinic for suspected ureteric colic; however, an abdominal ultrasound revealed only mild right‐sided pelvicalyceal fullness and was otherwise inconclusive. Her symptoms continued to worsen, leading to her current hospital admission.

## 3 Diagnostic Investigation and Differentials

On admission, the patient was febrile to 38.1°C, with a heart rate of 110 beats/min and blood pressure of 105/70 mmHg. Physical examination revealed significant tenderness to deep palpation in the right lower quadrant and suprapubic region, without rebound or guarding. A positive right psoas sign (pain on passive extension of the right hip) was elicited.

Initial laboratory investigations were notable for a marked leukocytosis of 18,500 cells/μL with an 88% neutrophil predominance, and a C‐reactive protein of 210 mg/L. Blood cultures drawn on admission later showed no growth. The primary differential diagnoses included a complex urinary tract infection with perinephric abscess, appendiceal abscess, tubo‐ovarian abscess, Crohn's disease, and a post‐procedural pelvic abscess.

**FIGURE 1:**
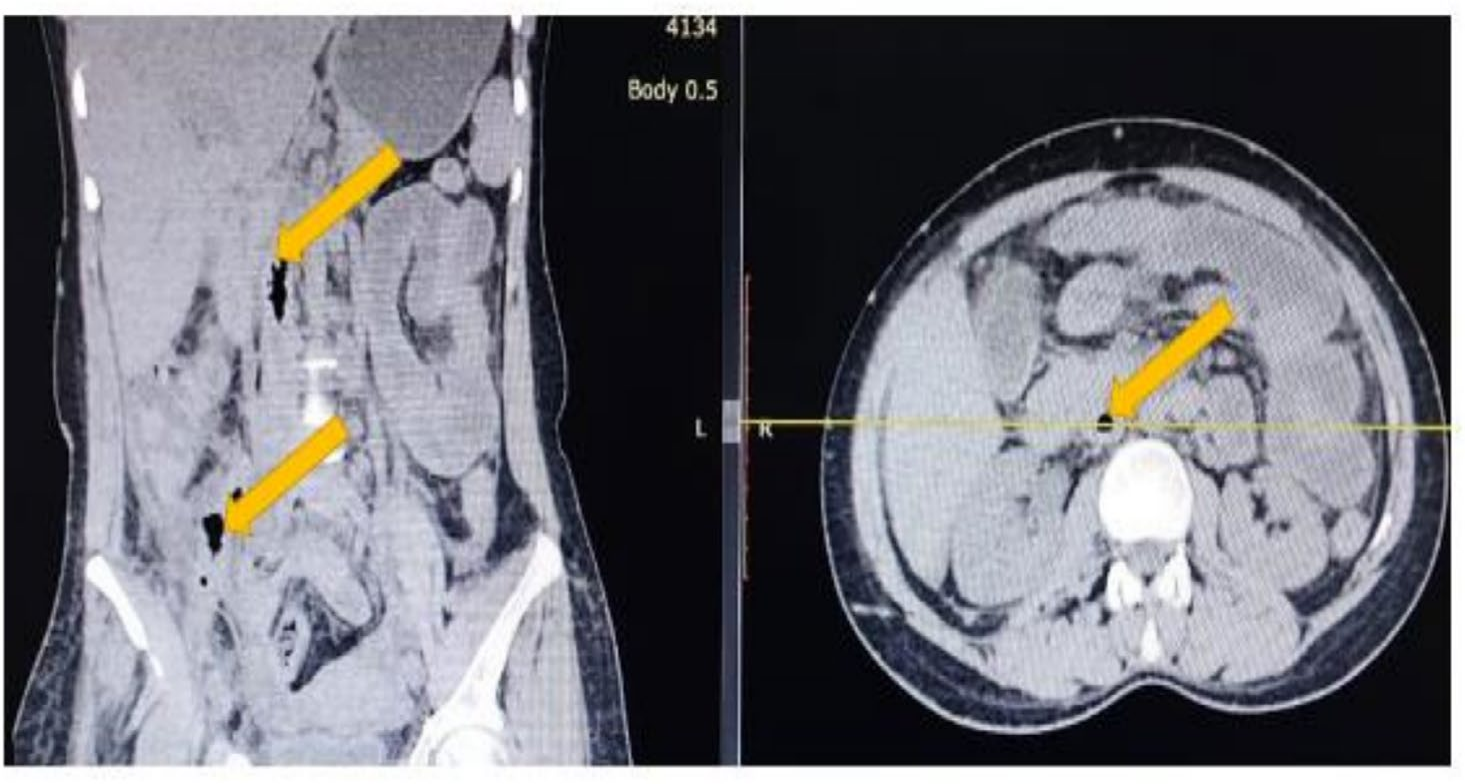
Bilateral psoas abscesses. A non‐contrast axial computed tomography (CT) image demonstrating large, complex collections within both psoas muscles, consistent with abscesses. The right‐sided collection is significantly larger and contains a substantial volume of gas.

**FIGURE 2:**
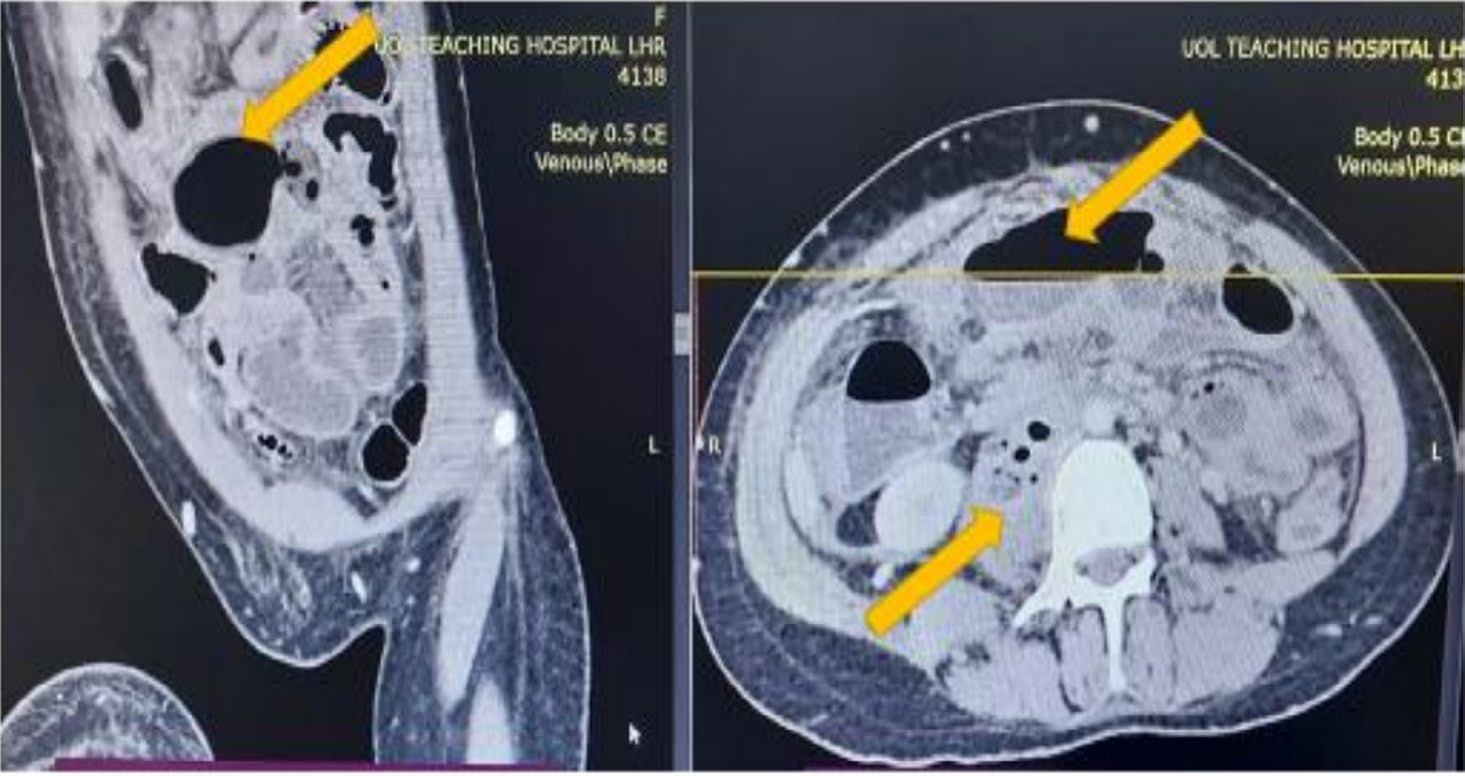
Pathognomonic finding of air in the inferior vena cava. A coronal reformatted contrast‐enhanced CT image clearly showing multiple locules of intraluminal gas within the inferior vena cava (IVC). This finding is pathognomonic for an enterovascular fistula. The large, rim‐enhancing right psoas abscess is also visible.

**FIGURE 3:**
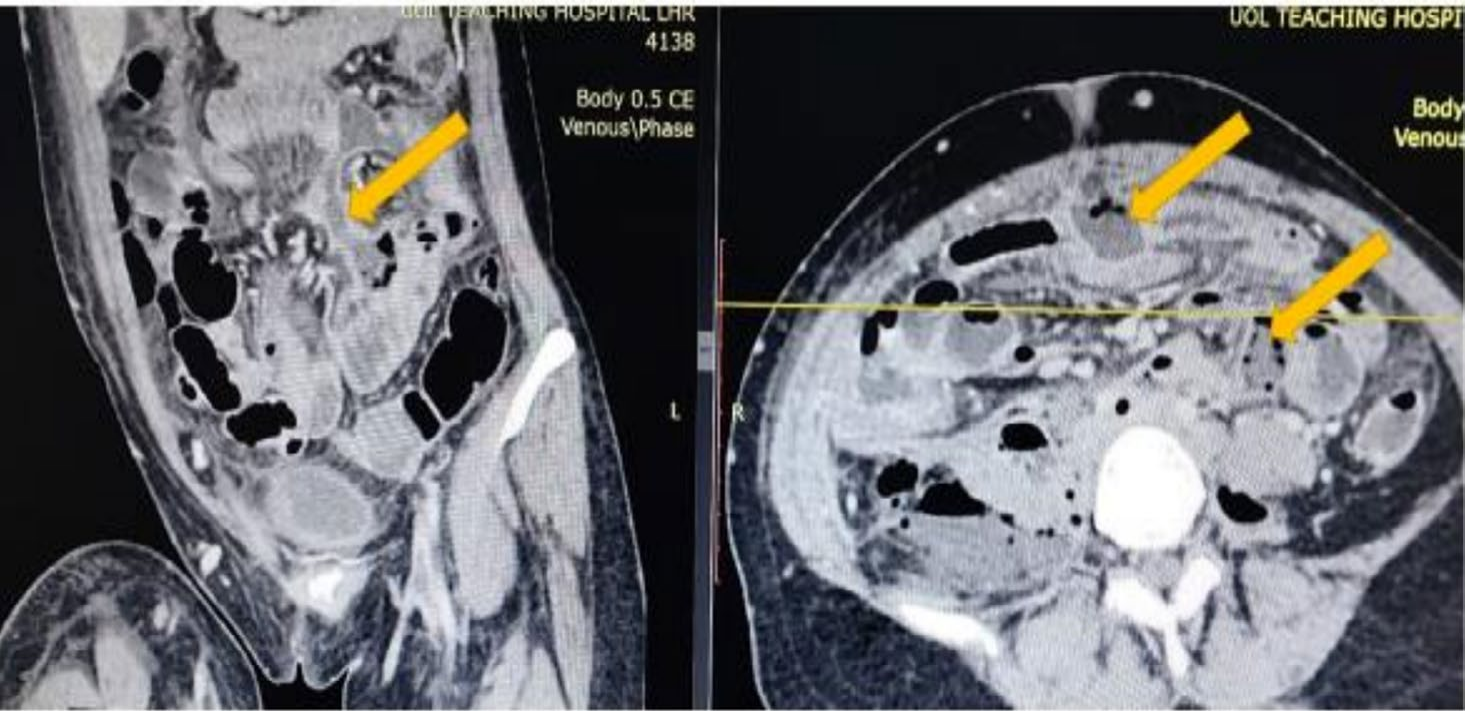
Communication between ileum and abscess cavity. An axial contrast‐enhanced CT image with oral contrast media. The image demonstrates a thickened and inflamed loop of the distal ileum immediately adjacent to the large right‐sided psoas abscess. A faint tract and inflammatory stranding can be seen between the bowel loop and the abscess cavity, confirming the site of the perforation and fistulous communication.

To further investigate, a contrast‐enhanced computed tomography (CECT) scan of the abdomen and pelvis was performed. The scan revealed multiple complex, rim‐enhancing intra‐abdominal collections, including large bilateral psoas abscesses (Figure 1), with the right‐sided abscess measuring 12 × 8 cm. The most critical and pathognomonic finding was the presence of multiple locules of free air within the lumen of the inferior vena cava (IVC), extending from the iliac confluence to the infrarenal segment, confirming an enterovascular fistula (Figure 2). A repeat CECT with oral contrast confirmed communication between a distal ileal loop and the right psoas abscess cavity (Figure 3).

## 4 Treatment, Follow‐Up, and Outcomes

The radiological diagnosis of an ileal perforation with psoas abscess and an ileo‐caval fistula constituted a surgical emergency. The patient was started on broad‐spectrum intravenous antibiotics (piperacillin‐tazobactam) and taken for an emergency exploratory laparotomy.

Intraoperatively, a contained 1.5 cm perforation was found on the anti‐mesenteric border of the terminal ileum, approximately 20 cm proximal to the ileocecal valve. The perforation led into a large, foul‐smelling retroperitoneal abscess that had infiltrated the right psoas muscle. Following evacuation of the abscess, a clear fistulous tract was identified extending from the abscess cavity directly into the anterolateral wall of the IVC. To address the presumed primary insult, the uterus was systematically inspected; it appeared grossly normal with no visible evidence of a persistent defect or scarring, suggesting the initial iatrogenic injury had sealed spontaneously. Intraoperative photography was deferred due to the critical nature of the patient's condition and the surgical team's focus on achieving rapid source control and hemodynamic stability.

A 15 cm segment of the perforated ileum was resected, and intestinal continuity was restored with a primary side‐to‐side anastomosis. For vascular control, the infrarenal IVC was carefully dissected, and proximal and distal control was obtained using atraumatic vascular clamps. The patient received a systemic bolus of 5000 units of unfractionated heparin approximately 3 min prior to vessel clamping to prevent intra‐caval thrombosis. The total clamp time was 14 min. The 4 mm fistulous opening in the IVC was repaired with a primary running suture of 5–0 polypropylene. The decision for primary suture repair was based on the small size of the defect and the healthy, non‐friable nature of the surrounding caval wall, which allowed for a tension‐free closure. This approach was preferred over patch angioplasty to avoid introducing prosthetic material into a grossly contaminated field, thereby minimizing the risk of subsequent graft infection.

The intra‐abdominal and psoas abscesses were then drained and copiously irrigated. Intraoperative fluid cultures subsequently grew *Escherichia coli* and *Bacteroides fragilis* , confirming a gastrointestinal source. Susceptibility testing confirmed sensitivity to the empiric piperacillin‐tazobactam regimen and also demonstrated susceptibility to a combination of ciprofloxacin and metronidazole. This data guided the de‐escalation to a targeted oral antibiotic course upon discharge. Histopathological examination of the resected ileal segment revealed transmural necrosis with acute and chronic inflammation consistent with a contained perforation, with no evidence of malignancy or inflammatory bowel disease.

The patient's postoperative course was managed in the intensive care unit for 48 h, where she remained hemodynamically stable. Her fever and leukocytosis resolved within 3 days. Postoperatively, the patient's thrombotic risk was carefully managed. For deep vein thrombosis (DVT) prophylaxis, she received subcutaneous enoxaparin 40 mg daily and wore sequential compression devices while hospitalized. Given the successful primary repair without stenosis and the absence of pre‐existing hypercoagulability, a long‐term course of therapeutic anticoagulation was deemed unnecessary. A planned surveillance protocol was instituted, beginning with a Doppler ultrasound prior to discharge to establish a new baseline and confirm IVC patency. She was discharged home on postoperative day 10 to complete a total 14‐day course of oral ciprofloxacin and metronidazole.

At her 6‐month follow‐up appointment, she was asymptomatic with full functional recovery. A repeat abdominal ultrasound with Doppler, as per the surveillance plan, confirmed the complete resolution of all abscess collections and demonstrated normal patency of the inferior vena cava with no evidence of thrombus or stenosis.

## 5 Discussion

This case details a catastrophic, delayed complication of uterine instrumentation, evolving from a contained ileal injury to a complex psoas abscess with a life‐threatening ileo‐caval fistula. While uterine perforation is a known risk of D&C, this unique constellation of sequelae presents a formidable diagnostic and management challenge [4].

The pathophysiological cascade likely began with an unrecognized uterine fundal perforation during the D&C, causing a direct, contained thermal or mechanical injury to an adjacent loop of terminal ileum. Rather than precipitating acute peritonitis, this insult likely manifested as a sealed micro‐perforation, initiating a localized, smoldering inflammatory process. Over the subsequent 3 months, this chronic, low‐grade infection evolved into a mature retroperitoneal abscess that expanded along the path of least resistance, eroding contiguously into both the right psoas muscle and the anterolateral wall of the Inferior Vena Cava (IVC).

To substantiate the rarity of this presentation, a literature review was conducted to identify reports of delayed (> 24 h) bowel injury after D&C. The search revealed a spectrum of delayed presentations, from subacute fecal peritonitis occurring within days to chronic, indolent courses lasting over a year with vague symptoms [5, 6]. As summarized in Table 2, these reports confirm that delayed bowel injury with subsequent abscess or fistula formation is a recognized, albeit rare, sequela of D&C [5, 6, 10]. However, our review confirms that the complete triad of a delayed ileal perforation, bilateral psoas abscesses, and direct fistulization into the inferior vena cava is previously unreported. This unique combination highlights the potential for contained septic processes to escalate into devastating vascular complications.

**TABLE 2:** Summary of published cases of delayed bowel injury with severe septic complications following D&C.

Author, year	Patient age	Time from D&C to presentation	Presenting symptoms	Bowel segment injured	Key complications	Surgical management	Outcome
Thanasa et al., 2025 [5]	62 Year‐Old	5 days	Acute abdomen, septic condition	Small bowel	Fecal peritonitis	Enterectomy, ileostomy	Survived after 22‐day ICU stay
Yu et al., 2024 [6]	35 Year‐Old	16 months	Non‐specific abdominal pain, oligomenorrhea	Ileum	Bowel herniation into uterine cavity	Laparoscopic enterolysis, hysterectomy, ileal resection & anastomosis	Not specified
Singh et al., 2020 [7]	28 Year‐Old	Not stated (diagnosed post‐delivery)	Passage of stool per vaginum	Ileum	Ileo‐uterine fistula	Fistula dismantling, bowel anastomosis, uterine repair	Uneventful recovery
Pereira et al., 2014 [8]	35 Year‐Old	4 days	Fevers, chills, malaise, right lower back, hip, and thigh pain	Not identified/not specified	Right iliopsoas abscess (without confirmed bowel perforation)	CT‐guided percutaneous drainage; Intravenous antibiotics	Uneventful recovery
Coffman, 2001 [9]	24 Year‐Old	10 days	Fever, abdominal pain, nausea, vomiting	Terminal Ileum	Delayed ileal perforation; Large pelvic abscess	Laparotomy, ileal resection, abscess drainage	Recovered
Present case	24 Year‐Old	3 months	Low‐grade fever, flank pain	Terminal Ileum	Bilateral psoas abscess, Ileo‐caval fistula	Ileal resection, abscess drainage, primary IVC repair	Full recovery

The management of a *recognized* uterine perforation typically involves either close hemodynamic observation or immediate diagnostic laparoscopy to rule out visceral injury [11]. This case, however, underscores the formidable diagnostic challenge of an *unrecognized* injury. The patient's insidious onset of non‐specific symptoms—low‐grade fever and progressive flank pain—was initially misattributed to a more common etiology, leading to a critical diagnostic delay. The central clinical message is that persistent, non‐specific abdominopelvic, flank, or back pain following any uterine instrumentation, even weeks to months post‐procedure, warrants a high index of suspicion for an occult bowel injury and mandates aggressive investigation with contrast‐enhanced cross‐sectional imaging.

This case also underscores the diagnostic challenge posed by an unrecognized uterine perforation. The patient's insidious onset of non‐specific symptoms led to a critical 3‐month diagnostic delay. While the management of a *recognized* perforation involves either observation or immediate laparoscopy, the greater clinical dilemma lies with an injury that is only *suspected* at the time of the procedure. In such instances where the surgeon suspects a perforation may have occurred but cannot definitively confirm or rule out visceral injury, a conservative yet vigilant approach is paramount. Admitting the patient for a 24‐h period of close inpatient observation, with serial abdominal examinations and monitoring of vital signs and inflammatory markers, is a prudent strategy to ensure early detection of evolving peritonitis or sepsis. This proactive step can prevent the catastrophic septic cascade seen in our patient.

In this instance, the Contrast‐Enhanced CT (CECT) findings were definitive. While the presence of psoas abscesses with a disproportionate amount of air strongly suggested a gastrointestinal source, the pathognomonic finding was the presence of free air within the lumen of the IVC. Intravascular gas is, in this case, an unambiguous sign of a communication between a gas‐forming medium and the vascular system, representing a true surgical emergency. This radiological sign signals an imminent risk of overwhelming sepsis or fatal systemic air embolism, mandating immediate surgical intervention without delay.

## 6 Conclusion

Delayed bowel perforation following D&C can evolve into complex, life‐threatening septic complications long after the index procedure. This case documents an exceptionally rare sequela: the formation of bilateral psoas abscesses and a direct ileo‐caval fistula presenting months after an apparently uncomplicated uterine instrumentation. The radiological finding of intravascular gas in this clinical setting is a pathognomonic sign of an enterovascular fistula. This finding represents a true surgical emergency, mandating immediate exploration to prevent the catastrophic consequences of systemic sepsis and air embolism.

## Author Contributions

**Faiza Farooq:** conceptualization, data curation, formal analysis, investigation, methodology, project administration, resources, supervision, validation, visualization, writing – original draft, writing – review and editing. **Bilal Aslam:** conceptualization, data curation, formal analysis, validation, visualization, writing – original draft, writing – review and editing. **Muhammad Hamza:** conceptualization, data curation, methodology, project administration, resources, software, writing – original draft, writing – review and editing. **Muhammad Saeed:** conceptualization, data curation, formal analysis, investigation, methodology, writing – original draft, writing – review and editing. **Khalil El Abdi:** conceptualization, data curation, formal analysis, methodology, project administration, resources, writing – original draft, writing – review and editing. **Fareena Ambreen:** conceptualization, data curation, formal analysis, methodology, project administration, writing – original draft, writing – review and editing. **Abdul Eizad Asif:** conceptualization, data curation, formal analysis, methodology, project administration, writing – original draft, writing – review and editing. **Fazeela Bibi:** conceptualization, data curation, methodology, project administration, visualization, writing – original draft, writing – review and editing. **Youssef Dadouche:** conceptualization, data curation, formal analysis, investigation, methodology, project administration, writing – original draft, writing – review and editing. **Zaid Saimeh:** conceptualization, data curation, investigation, methodology, project administration, resources, supervision, validation, visualization, writing – original draft, writing – review and editing. **Umama Alam:** conceptualization, data curation, formal analysis, investigation, methodology, project administration, resources, software, writing – original draft, writing – review and editing. **Said Hamid Sadat:** writing – original draft, writing – review and editing.

## Funding

The authors have nothing to report.

## Ethics Statement

The authors have nothing to report.

## Consent

Written Informed consent was taken from the patient to publish this case report.

## Conflicts of Interest

The authors declare no conflicts of interest.

## Data Availability

The data were taken from a patient who presented to our hospital; all data and references are publicly available on databases such as Pub‐med and Google Scholar.
